# Imaging Mass Spectrometry of Isotopically Resolved Intact Proteins on a Trapped Ion-Mobility Quadrupole Time-of-Flight Mass Spectrometer

**DOI:** 10.1021/acs.analchem.3c05252

**Published:** 2024-03-22

**Authors:** Dustin R. Klein, Emilio S. Rivera, Richard M. Caprioli, Jeffrey M. Spraggins

**Affiliations:** † Mass Spectrometry Research Center, Vanderbilt University, Nashville, Tennessee 37235, United States; ‡ Department of Biochemistry, Vanderbilt University, Nashville, Tennessee 37235, United States; § Department of Chemistry, Vanderbilt University, Nashville, Tennessee 37235, United States; ∥ Department of Medicine, Vanderbilt University, Nashville, Tennessee 37235, United States; ⊥ Department of Pharmacology, Vanderbilt University, Nashville, Tennessee 37235, United States; ○ Department of Cell and Developmental Biology, Vanderbilt University, Nashville, Tennessee 37235, United States; □ Department of Pathology, Microbiology, and Immunology, Vanderbilt University Medical Center, Nashville, Tennessee 37235, United States

## Abstract

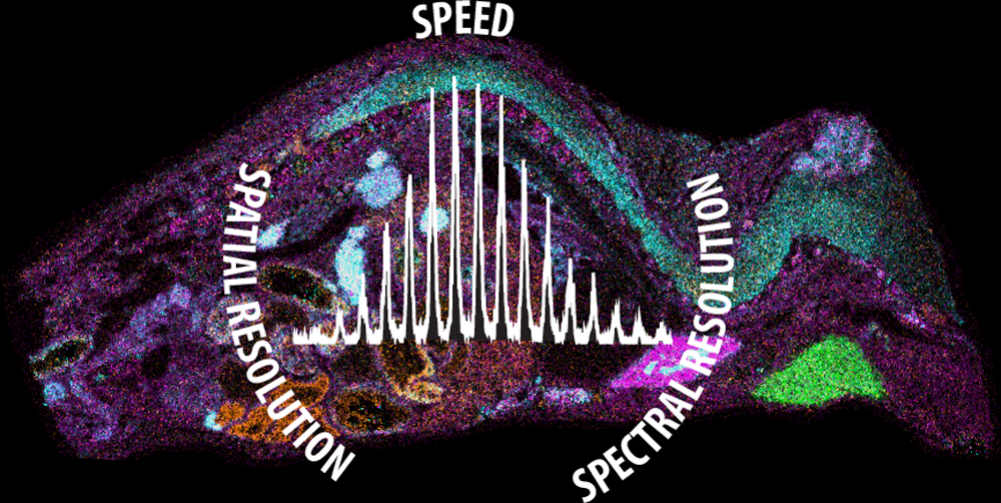

In this work, we demonstrate rapid, high spatial, and high spectral resolution imaging of intact proteins by matrix-assisted laser desorption/ionization (MALDI) imaging mass spectrometry (IMS) on a hybrid quadrupole-reflectron time-of-flight (qTOF) mass spectrometer equipped with trapped ion mobility spectrometry (TIMS). Historically, untargeted MALDI IMS of proteins has been performed on TOF mass spectrometers. While advances in TOF instrumentation have enabled rapid, high spatial resolution IMS of intact proteins, TOF mass spectrometers generate relatively low-resolution mass spectra with limited mass accuracy. Conversely, the implementation of MALDI sources on high-resolving power Fourier transform (FT) mass spectrometers has allowed IMS experiments to be conducted with high spectral resolution with the caveat of increasingly long data acquisition times. As illustrated here, qTOF mass spectrometers enable protein imaging with the combined advantages of TOF and FT mass spectrometers. Protein isotope distributions were resolved for both a protein standard mixture and proteins detected from a whole-body mouse pup tissue section. Rapid (∼10 pixels/s) 10 μm lateral spatial resolution IMS was performed on a rat brain tissue section while maintaining isotopic spectral resolution. Lastly, proof-of-concept MALDI-TIMS data was acquired from a protein mixture to demonstrate the ability to differentiate charge states by ion mobility. These experiments highlight the advantages of qTOF and timsTOF platforms for resolving and interpreting complex protein spectra generated from tissue by IMS.

## Introduction

Untargeted biomolecular imaging of tissues has largely been driven by the advancement of imaging mass spectrometry (IMS).^1−5^ During a typical IMS experiment, analytes are sampled and ionized directly from tissue surfaces along a coordinate plane, detected by a mass spectrometer, and visualized as ion heat maps according to ion abundances.^2^ The lateral spatial resolution of an ion image is reported as the tissue^3^ area used to represent a single mass spectrum, i.e., one spectrum per pixel. IMS has been used to map distributions of metabolites, lipids, peptides, proteins, and glycans, and is complementary to histological methods (stained and antibody-based imaging).^2,6−8^ Proteins can be analyzed either intact or as peptides generated after on-tissue enzymatic digestion.^6,9,10^ While on-tissue digestion strategies expand the range of detectable proteins, biologically relevant proteoform information (e.g., post-translational modifications, protein sequence truncations, point mutations, etc.) is potentially lost.^11,12^ Alternatively, proteoform information is preserved during intact protein analysis; however, the size of detectable proteins is expected to be limited with this approach.

Matrix-assisted laser desorption/ionization (MALDI), which generates low-charge state ions at relatively high mass-to-charge (*m*/*z*) ratios, is the most commonly used ionization method for intact protein IMS. Therefore, intact protein MALDI IMS experiments are typically performed using time-of-flight (TOF) mass spectrometers owing to their theoretically unlimited *m*/*z* range and high sensitivity. Novel sample preparation techniques and high mass detectors on TOF instruments have enabled the detection of proteins ≥50 kDa using TOF platforms.^13−15^ Lui et al. recently reported the use of caffeic acid to detect intact proteins from tissue at *m*/*z* ∼ 190000.^14^ In addition, advances in MALDI-TOF instrumentation and laser optics have enabled rapid acquisition of spectra from increasingly small sampling regions;^16−19^ ion images acquired at a rate of up to 30 pixels/s with a lateral spatial resolution of 5 μm, have been reported.^18^ However, during tissue analysis, the limited mass resolving power of TOF instruments, especially with detection in linear mode, results in convoluted spectra from which few accurate protein and proteoform intact masses can be determined.

Implementing MALDI on Fourier transform-ion cyclotron resonance (FT-ICR) and Orbitrap mass spectrometers has enabled high resolution and mass accuracy measurements for both intact endogenous proteins from tissue and protein standards.^11,19−24^ Historically, FT-ICR and Orbitrap instruments have had lower and narrower *m*/*z* ranges by comparison to TOF mass spectrometers, which has restricted intact protein analysis by MALDI on high-resolution instruments. Strategies to overcome *m*/*z* range limitations have included manipulation of instrument source pressure and tuning of ion optics for increased ion transmission efficiency,^22^ and use of novel matrices to achieve higher charge state ions.^24^ The advent of an extended *m*/*z* range Orbitrap mass spectrometer with an upper range limit of *m*/*z* 80000 also holds promise for intact protein IMS experiments, as was recently demonstrated for spatial mapping of histone proteoforms in human kidney tissue.^25,26^ While the mass spectral characteristics of FT-ICR and Orbitrap mass analyzers are attractive, it is crucial to consider the inverse relationship between *m*/*z* and resolving power when performing MALDI IMS on FT-MS systems.^27^ To achieve resolving powers capable of providing isotopic resolution at high *m*/*z* values, increasingly long scan times (i.e., time-domain transient lengths of ≫1s) are required.^22^ During a MALDI IMS experiment, where tens to hundreds of thousands of spectra are collected, data acquisition times can exceed 24 h. Experiments are often conducted at lateral spatial resolutions ≥75 μm or lower mass resolving powers to mitigate the time cost associated with MALDI IMS on FT-MS instruments.^22^ Computational approaches are also being developed to improve protein imaging performance. Image fusion workflows combining data sets from MALDI-TOF and MALDI FT-ICR mass spectrometers have been developed to capitalize on the benefits of each instrument, for example.^28^

The future of intact protein IMS relies on developing instrumentation that combines speed, high lateral spatial resolution, and high spectral resolution. Hybrid quadrupole-TOF (qTOF) mass spectrometers possess each of these desirable characteristics and have the potential to combine the benefits of both FT-MS and TOF platforms. Unlike FT-MS systems where instrument resolving power decreases with increasing *m*/*z*, TOF and qTOF mass spectrometers have relatively constant resolving powers across the *m*/*z* range. Therefore, increased instrument scan times are unnecessary to achieve high mass resolution at high *m*/*z*. A recently introduced qTOF mass spectrometer equipped with a MALDI source and trapped ion mobility spectrometry (TIMS) was shown to provide rapid spectral acquisition rates (≥20 Hz), high lateral spatial resolution (10 μm), and spectral resolution greater than 40000 for lipid imaging.^29^ The present work demonstrates the advantages of using this mass spectrometer in qTOF-mode for rapid MALDI IMS of isotopically resolved proteins at high lateral spatial resolution. In addition, proof-of-concept TIMS data for MALDI-generated protein standards shows the potential for further spectral deconvolution during protein IMS.

## Experimental Section

More detailed methods can be found in the Supporting Information. Briefly, all experiments were conducted in positive ion mode on a MALDI timsTOF Pro mass spectrometer (Bruker Daltonics, Billerica, MA, U.S.A.) equipped with a SmartBeam 3D 10 kHz frequency tripled Nd:YAG laser (355 nm) operated with beam scanning on.^29^ This instrument will be referred to as a timsTOF fleX throughout this work. Spectra of spotted protein standards in “qTOF”-mode, where no ion mobility separation was performed, and TIMS-mode were acquired with a pixel size of 50 μm × 50 μm. IMS of the whole-body mouse pup was also performed similarly. IMS of the rat brain tissue section was performed with a pixel size of 10 μm × 10 μm. For optimal transmission of protein ions, the following instrument parameters were used: ion transfer time = 300 μs; prepulse storage = 50 μs; collision RF = 4000 V; collision energy = 10 eV; ion energy in the quadrupole = 5 eV; TIMS Funnel 1 RF = 500 Vpp; TIMS Funnel 2 RF = 475 Vpp. An ion mobility separation time of 200 ms and a reduced mobility (1/K_0_) range of 0.8 to 5.0 were used.

## Results

The feasibility of high *m*/*z* detection and calibration was assessed here via MALDI-generated ions using red phosphorus, an analyte that produces a predictable series of clusters upon laser irradiation. The scan range of the instrument was set to *m*/*z* 2000–20000 (Figure S1). The prepulse storage and ion transfer time were adjusted to 50 and 300 μs, respectively, to allow for the transmission of higher *m*/*z* ions. Ions were detected up to *m*/*z* ∼ 17000, confirming that this system can be tuned to detect high *m*/*z* ions and that red phosphorus ions are a suitable calibration standard for this platform over a wide mass range. Table S1 shows the list of detected red phosphorus ions that were used for calibration. MALDI has been previously utilized on the timsTOF fleX for analyzing metabolites,^30^ lipids,^29^ and glycans,^31^ where ions were detected up to *m*/*z* 4000. The detection of ions with *m*/*z* ∼ 17000 far exceeds these previous results and is comparable to work published by Fernandez-Lima and co-workers, where they used a modified timsTOF Pro to detect ESI-generated oligomer ions from a calibration standard up to *m*/*z* 19000.^32^

**Figure 1:**
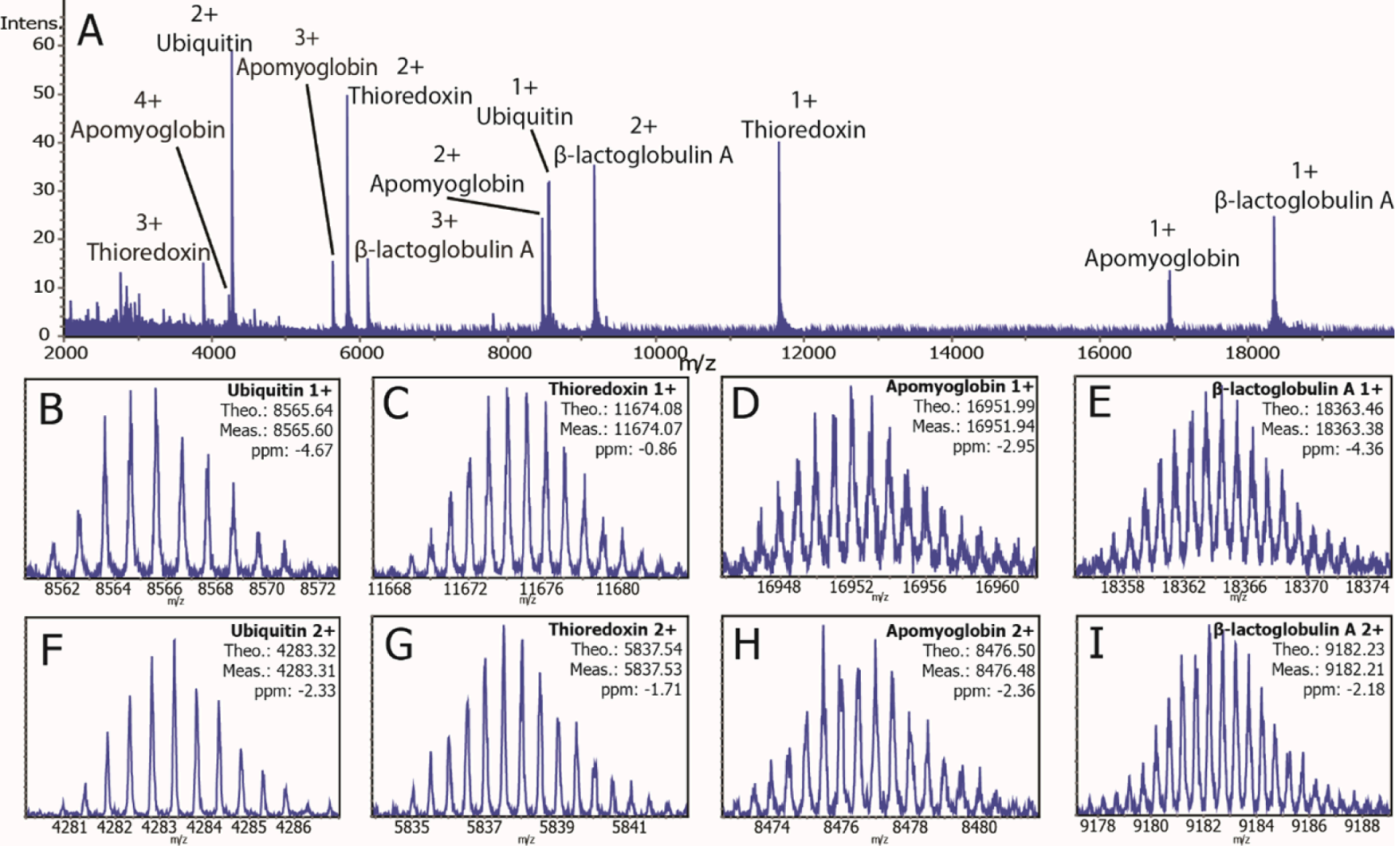
MALDI mass spectrum of a protein standard mixture composed of ubiquitin (8.6 kDa), thioredoxin (11.6 kDa), apomyoglobin (16.9 kDa), and β-lactoglobulin (18.3 kDa) (A) with expanded insets of the [M + H]^+^ (B–E) and [M + 2H]^2+^ (F–I) charge states. A range of charge states are detected for each protein, and all protein ions in the [M + H]^+^ and [M + 2H]^2+^ can be isotopically resolved.

To assess instrument performance for intact proteins, MALDI spectra were collected for a protein mixture containing ubiquitin, thioredoxin, apomyoglobin, and β-lactoglobulin with a range of *m*/*z* 2000–20000 (Figures 1 and S2 and Table S2). Data represented in Figures 1 and S2 are averages of 50 spectra. A range of charge states are observed for each protein, including the [M + H]^+^ ion of β-lactoraglobulin detected at over *m*/*z* 18000. Like previous work conducted by Prentice et al. on a MALDI FT-ICR mass spectrometer, the influence of instrument source pressure on mass range and protein charge state intensities was evaluated by adjusting the TIMS tunnel pressure (determined by the TIMS Tunnel In Pressure instrument reading, Figure S2). For ease of spectral comparison, the spectrum from Figure 1a showing the MALDI mass spectrum at a TIMS tunnel pressure of 1.5 mbar is included in Figure S2 as Figure S2g. Similar to the results reported by Prentice et al. for decreasing ion funnel pressure, reducing TIMS tunnel pressure increases higher *m*/*z* ion intensity. For example, at a TIMS tunnel pressure of 2.6 mbar, which is considered the normal operating pressure for typical proteomic and lipidomic workflows, the [M + H]^+^ charge states of apomyoglobin and β-lactoglobulin at *m*/*z* 16000 and *m*/*z* 18000, respectively, are nearly indistinguishable from the instrument noise. With decreasing TIMS tunnel pressure, a gradual increase in signal intensity is observed for singly-charged ions. These results are consistent with results recently presented in a Bruker Daltonics technical note.^33^ Consequently, all qTOF-only experiments were conducted at 1.5 mbar, the lowest TIMS tunnel pressure setting. Figure 1b–i contain a series of expanded spectra for the [M + H]^+^ and [M + 2H]^2+^ charge states of each protein in the protein standard mixture. Protein signals are isotopically resolved for all charge states, as expected for a constant instrument resolving power of ∼40000 across the *m*/*z* range. In addition, calculated ppm error values for the most intense isotopes of the [M + H]^+^ and [M + 2H]^2+^ ions for each protein are <5 ppm. (Sequences for protein standards are provided in Table S2.) Notably, this data is comparable to that collected on an FT-ICR mass spectrometer during a MALDI protein imaging experiment.^22^

**Figure 2:**
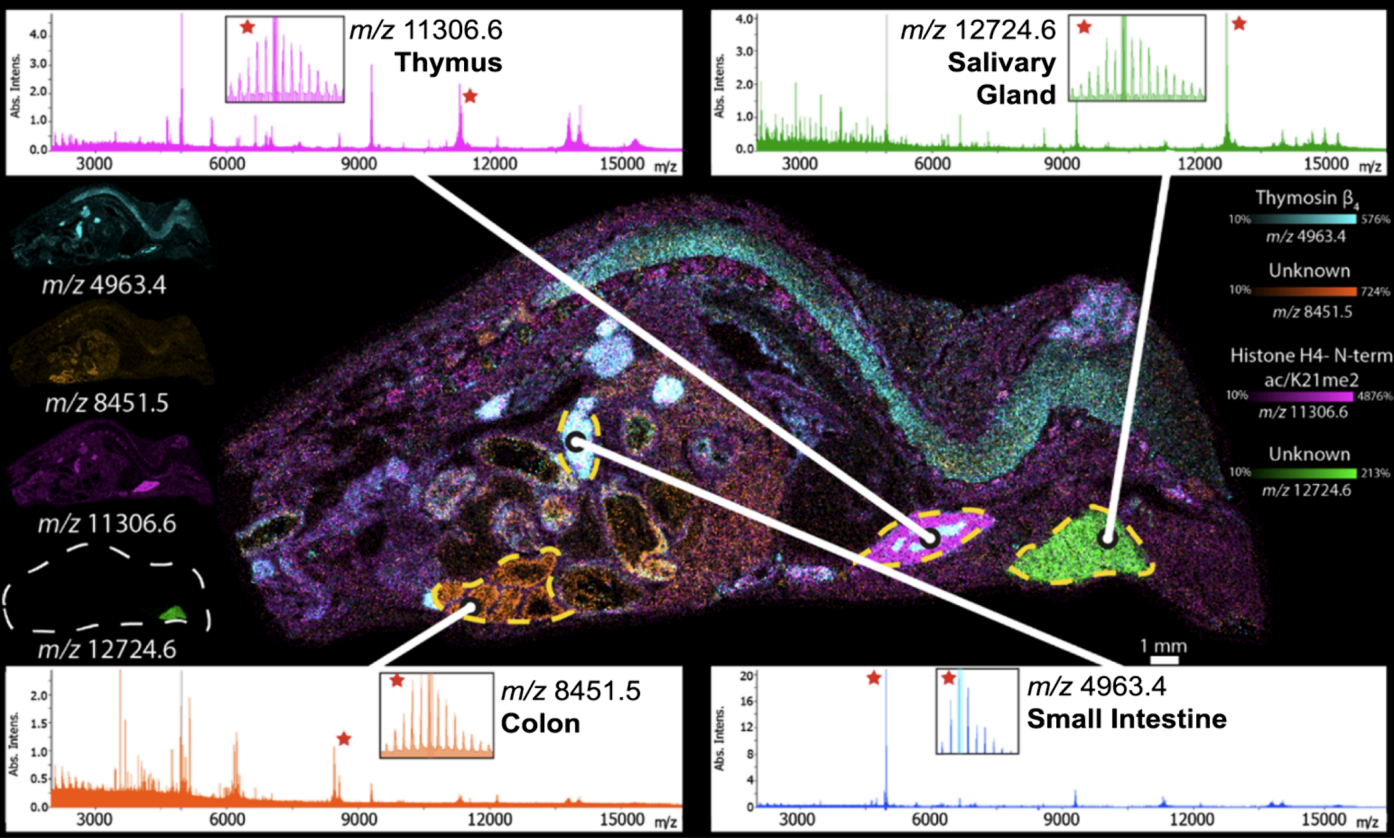
Intact protein IMS performed on a mouse pup tissue. Individual and overlaid ion images of *m*/*z* 4963.4, *m*/*z* 8451.5, *m*/*z* 11306.6, and *m*/*z* 12724.6 show distinct localization of proteins to anatomical features. Averaged mass spectra are shown for regions surrounded by a dotted yellow line. Peaks used to generate ion images are labeled with a red star, and expanded insets are used to show protein ions with resolved isotope distributions.

The high mass resolution, accurate mass MALDI spectra collected for protein standards confirms the suitability of the timsTOF fleX for intact protein analysis. Using the same method, protein IMS was performed on a tissue section of a whole-body mouse pup with a lateral spatial resolution of 50 μm. Figure 2a depicts individual and overlaid ion images for *m*/*z* 4963.4, *m*/*z* 8451.5, *m*/*z* 11306.6, and *m*/*z* 12724.6. Ion images are generated from the most intense individual peak from each isotope distribution. Averaged spectra extracted from specific anatomical features (outlined with dotted yellow lines) are shown in Figure 2b–e. Insets within Figure 2b–e show expanded spectra for each imaged *m*/*z* value and confirm baseline isotopic resolution was achieved across the measured *m*/*z* range. The ions of *m*/*z* 4963.4 and *m*/*z* 11306.6 were identified as thymosin-β4 and histone H4 based on mass accuracy. An average spectrum across the entire mouse pup section is shown in Figure S3, and additional ion images for other *m*/*z* values are shown in Figure S4. Although the overall average spectrum appears sparse (Figure S3), it is noted that the spectra averaged from selected tissue regions (Figure 2b–e) highlight the richness and complexity of the proteins detected. This is common for protein imaging experiments because many proteins have very distinct localizations being detected in only a small fraction of the total number of pixels collected, in this case, across the whole-body image. This leads to many species being “averaged out” when looking at the overall spectrum. In these experiments, we detect hundreds of proteins. The high plexity of MALDI IMS, the potential to map post-translational modifications, and the ability to perform the analysis without any antibody markers are significant advantages over traditional immunohistochemical approaches.

**Figure 3:**
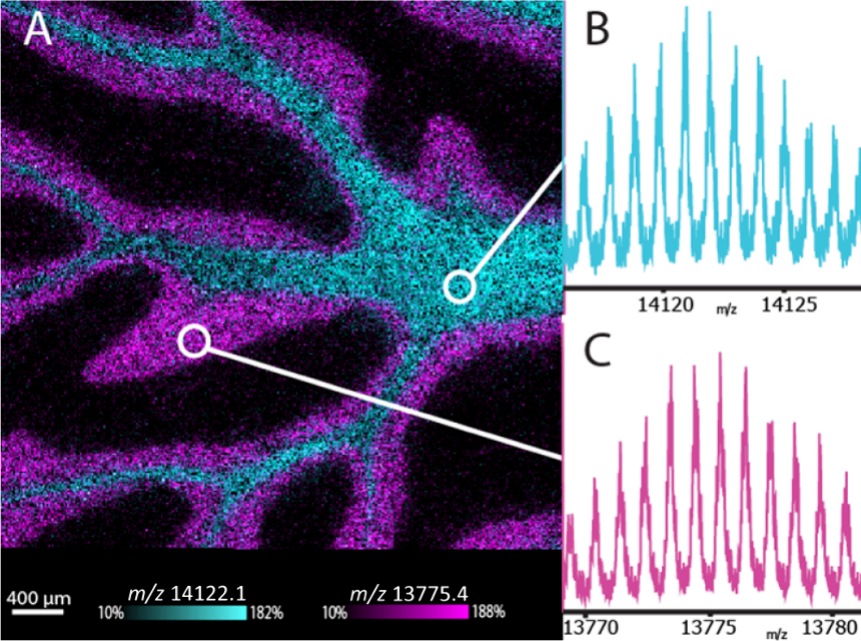
Ten μm protein IMS of rat brain tissue. Overlaid ion images of *m*/*z* 14122.1 and *m*/*z* 13775.4 show that high lateral spatial resolution is possible on the timsTOF fleX (A). Expanded mass spectra of protein isotope distributions (B, C) confirm that high lateral spatial resolution can be performed without compromising mass spectral resolution. Mass spectra are averages generated from regions of interest drawn within each morphological structure (7914 pixels for A and 9507 pixels for B).

Previous work on the timsTOF fleX demonstrated that lipid imaging could be conducted with a lateral spatial resolution of 10 μm.^29^ To evaluate the potential for imaging of intact proteins at 10 μm, protein IMS was performed on an axial rat brain tissue section (Figure 3). Overlaid ion images of *m*/*z* 13775.4 and *m*/*z* 14122.1 localizing to the granular cell layer and white matter, respectively, confirm that 10 μm protein IMS data can be collected on the timsTOF fleX. To compensate for the lower signal intensities that result from sampling smaller areas, ion images here were generated from entire isotope distributions. The ion image in Figure 3 contains 178590 pixels and took 4.6 h to collect, corresponding to a scan rate of ∼10 pixels per second. For comparison, to theoretically collect ∼178000 pixels on a 15 T FT-ICR mass spectrometer with a spectral resolving power of 40000 at *m*/*z* ∼ 14000 would take approximately 145 h (eqns S1 and S2). Collection of MALDI IMS data on a qTOF mass spectrometer, therefore, can provide a >30× improvement in time savings without sacrificing instrument resolving power compared to FT-MS platforms.

**Figure 4:**
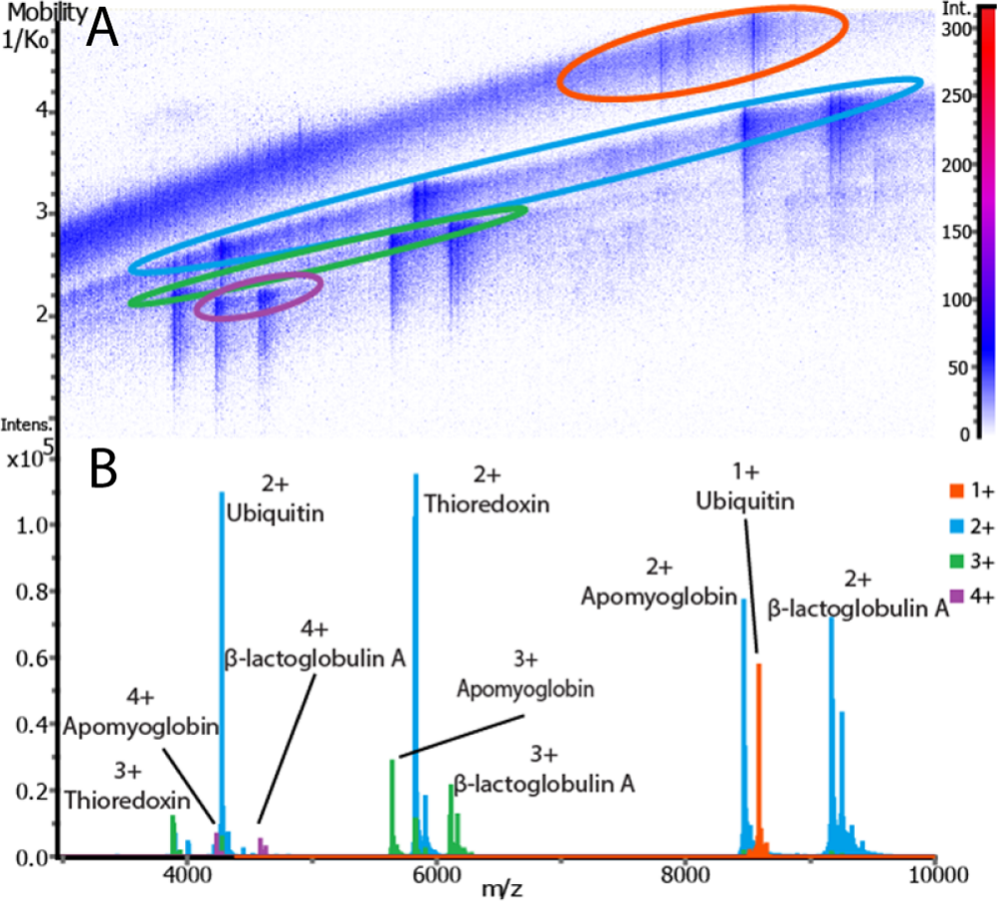
Integration of TIMS allows for the separation of intact protein ions along charge state trendlines. The *m*/*z*–1/K_0_ heat map (A) contains a series of colored circles corresponding to charge state trendlines. Spectra extracted from the *m*/*z*–1/K_0_ heat map (B) are overlaid and color-coded according to the circled regions in part A.

With the analysis of increasingly complex mixtures, separation before mass analysis can be beneficial. Incorporating ion mobility into IMS experiments to provide a dimension of separation orthogonal to *m*/*z* has become increasingly popular. For example, TIMS has been utilized to separate lipid isobars during MALDI IMS analysis,^29,34^ and field asymmetric ion mobility spectrometry (FAIMS) has been used during LESA-IMS to increase the number of detectable proteins.^35^Figure 4a shows a heat map of *m*/*z* vs the inverse of the reduced mobility (1/K_0_) obtained during MALDI-TIMS analysis of the protein standard mixture. Consistent with previously reported ion mobility data, TIMS provides charge state separation in the form of trendlines, with higher charge state ions having lower 1/K_0_ values (Figure 4a).^32^Figure 4b shows overlaid mass spectra extracted from each region circled in the *m*/*z*–1/K_0_ heat map. When performing TIMS separations, the highest *m*/*z* values detected were around *m*/*z* 9000. In the future, adjusting ion optic RF frequencies and additional instrument tuning may provide a means to increase ion transmission efficiency for higher *m*/*z* ions during TIMS experiments.

## Conclusions

This work demonstrates that untargeted protein IMS performed on a qTOF mass spectrometer enables rapid, high lateral spatial resolution imaging without sacrificing spectral resolving power. In addition, preliminary studies showed the potential for TIMS to simplify analysis by separating proteins based on charge state. Despite the clear benefits of protein IMS on a qTOF mass spectrometer, protein identification remains challenging. While top-down tandem mass spectrometry (MS/MS) is typically the method of choice for intact protein characterization, two instrument attributes must be considered for MALDI-generated ions on a qTOF: (1) the upper *m*/*z* limit for ion isolation and (2) the available ion activation methods. Adjustment of ion optic and quadrupole RF frequencies can potentially extend the upper *m*/*z* limit of ion isolation. After ion isolation, producing informative fragment ions during MS/MS is crucial to analyte identification. Low-energy collisional activation, the most commonly used ion activation on qTOF instruments, yields few informative fragment ions for low-charge state ions. Alternative ion activation methods, including ultraviolet photodissociation (UVPD), have previously been shown to provide increased protein sequence coverage for singly charged ions generated by MALDI.^20^ Future implementation of UVPD on a MALDI-qTOF mass spectrometer may benefit *in situ* protein identification, providing much needed capabilities for untargeted, discovery-based spatial proteomics analysis. In summary, the reduced data acquisition time afforded by performing protein MALDI IMS on a qTOF mass spectrometer lowers the barrier to routine collection of high spatial and spectral resolution protein IMS data. Importantly, developing methods using a common MALDI qTOF platform provides a starting point for the entire IMS community to advance the field of spatial proteomics.

## Supporting Information Available

Figure S1: MALDI mass spectrum of red phosphorus; Figure S2: MALDI mass spectra of the protein standard as varying TIMS Funnel In pressures; Figure S3: MALDI mass spectrum averaged across the entire mouse pup tissue section; Figure S4: Mouse pup ion images; Table S1: List of red phosphorus ions used for instrument calibration; Table S2: Table of protein standards, including amino acid sequences; Equation S1: Equation for theoretical spectral acquisition time on a 15T FT ICR; Equation S2: Equation for theoretical total aqusution time for a 170000 pixel MALDI IMS image on a 15T FT-ICR (PDF)
