# Supplementary material for: Imaging Mass Spectrometry of Isotopically Resolved Intact Proteins on a Trapped Ion-Mobility Quadrupole Time-of-Flight Mass Spectrometer
Source: Anal Chem. 2024 Mar 22;96(13):5065–70. doi: 10.1021/acs.analchem.3c05252 (PMC10993197; doi:10.1021/acs.analchem.3c05252)
Supplement: Supplementary file 1 — ac3c05252_si_001.pdf [file ac3c05252_si_001.pdf]

Imaging mass spectrometry of isotopically-resolved intact proteins on a trapped ion-mobility quadrupole time-of-flight mass spectrometer

Dustin R. Klein[1,2+], Emilio S. Rivera[1,2+], Richard M. Caprioli[1,2,3,4,5], Jeffrey M. Spraggins[1,2,3,6,7]*

[1]Mass Spectrometry Research Center, Vanderbilt University, Nashville, TN 37235, USA
[2]Department of Biochemistry, Vanderbilt University, Nashville, TN 37235, USA
[3]Department of Chemistry, Vanderbilt University, Nashville, TN 37235, USA
[4]Department of Medicine, Vanderbilt University, Nashville, TN 37235, USA
[5]Department of Pharmacology, Vanderbilt University, Nashville, TN 37235, USA
[6]Department of Cell and Developmental Biology, Vanderbilt University, Nashville, TN 37235, USA
[7]Department of Pathology, Microbiology, and Immunology, Vanderbilt University Medical Center, Nashville, TN 37235, USA

[+]Authors contributed equally

*Corresponding Author: Jeffrey M. Spraggins, jeff.spraggins@vanderbilt.edu

	Content	Page
**Experimental Details**		S-2
**Figure S1.**	MALDI mass spectrum of red phosphorus	S-3
**Figure S2.**	MALDI mass spectra of the protein standard as varying TIMS Funnel In pressures	S-4
**Figure S3.**	MALDI mass spectrum averaged across the entire mouse pup tissue section	S-5
**Figure S4.**	Mouse pup ion images	S-6
**Table S1.**	List of red phosphorus ions used for instrument calibration	S-7
**Table S2.**	Table of protein standards including amino acid sequences	S-8
**Equation 1.**	Equation of theoretical spectral aqcuisition time on a 15T FT-ICR	S-9
**Equation 2.**	Equation used to calculate the total acquisition time for a MALDI image	S-9
**Reference**		S-10

Experimental Details

Materials: 2,5-dihydroxyacetophenone (DHA) matrix, red phosphorus, and the protein standards ubiquitin (8.6 kDa), thioredoxin (11.6 kDa), apomyoglobin (16.9 kDa), and β-lactoglobulin (18.3 kDa), toluene, and ammonium hydroxide were purchased from Sigma-Aldrich (St. Louis, MO). (E)-4(2,5-dihdroxphenyl)but-3-en-2one (2,5-cDHA) was synthesized in-house.[1] Tissues were purchased from BioIVT (Westbury, NY). Indium-tin-oxide (ITO)-coated microscope slides were purchased from Delta Technologies (Loveland, CO). Agilent Tuning mix was purchased from Agilent (Santa Clara, CA). Water, chloroform, acetonitrile, ethyl acetate, acetic acid, and formic acid were purchased from Fisher Scientific (Waltham, MA). Rat brain tissue was purchased from Pel-Freeze Biologicals (Rogers, AR) and stored at −80 °C until analysis.

Sample preparation: For protein standard analysis, a mixture of protein standards [ubiquitin (2 pmol/uL), apomyoglobin (4 pmol/uL), thioredoxin (6 pmol/uL), β-lactoglobulin (8 pmol/uL)] in water were combined 1:1 with 15 mg/mL DHA in ACN/H_2O (90:10) with 0.1% formic acid. A series of 1 uL aliquots were deposited onto an MTP AnchorChip MALDI target (Bruker Daltonik, Bremen, Germany) and dried. For imaging experiments, all tissues were sectioned using a CM3050S cryostat from Leica Microsystems (GmbH, Wetzlar, Germany) and thaw-mounted on ITO slides. Rat brain tissues were sectioned at 10 µm thickness. A one-week-old C57BL/6 control mouse pup was snap-frozen at −80 °C, shaved over dry ice, and cryosectioned at a 20 µm thickness. Samples were then subjected to an ethanol submersion sequence as follows: 70%, 90%, 95%, and 100% ethanol for 30 s each. Next, samples were submerged in Carnoy's solution containing chloroform, methanol and acetic acid (6:3:1) for 3 min, followed by 90% ethanol, and finally pure water for 30 s. Tissues were coated with 2,5-cDHA matrix following a previously published method.[1] Briefly, a 3 mg/mL solution of cDHA dissolved in ethyl acetate and toluene (1:1) was applied, followed by a second application of 9 mg/mL solution dissolved in ACN/H_2O (3:7) with 1% TFA and 0.5% ammonium hydroxide, each using a TM Sprayer (HTX Technologies, LLC, Chapel Hill, NC). Tissues were rehydrated by suspending samples over 1 mL of 50 mM acetic acid for 3 min at 37° C.

Mass Spectrometry and Ion Mobility: All experiments were conducted in positive ion mode on a prototype MALDI timsTOF Pro mass spectrometer (Bruker Daltonik, Bremen, Germany) equipped with a SmartBeam 3D 10 kHz frequency tripled Nd:YAG laser (355 nm) operated with beam scanning on.[2] This instrument is the predecessor of the now commercialized Bruker timsTOF fleX. The primary components are the same as the commercial platform with negligible performance differences between the platforms. As such, we will be referred to as a timsTOF fleX throughout this work. The resolving power of this instrument is ~40,000. Spectra of spotted protein standards in both "qTOF"-mode, where no ion mobility separation was performed, and TIMS-mode were acquired with a pixel size of 50 µm x 50 µm with 1000 laser shots per pixel. IMS of the whole-body mouse pup was also performed at a pixel size of 50 µm x 50 µm with 1,000 laser shots per pixel. IMS of the rat brain tissue section was performed with a pixel size of 10 µm x 10 µm with 750 laser shots per pixel. For optimal transmission of protein ions, the following instrument parameters were used: ion transfer time = 300 µs; pre-pulse storage = 50 µs; collision RF = 4000 V; collision energy = 10 eV; ion energy in the quadrupole = 5 eV; TIMS Funnel 1 RF = 500 Vpp; TIMS Funnel 2 RF = 475

Vpp. For analysis of the protein standard mixture using TIMS, calibration was performed using Agilent Tuning mix. To achieve a reduced mobility ($1/K_0$) range of 0.8 to 5.0, calibration was performed after a series of incremental decreases in the TIMS Tunnel In pressure. An ion mobility separation time of 200 ms and a reduced mobility ($1/K_0$) range of 0.8 to 5.0 were used. Bruker Data Analysis software and SCiLS Lab Version 2020 (Bruker Daltonics, Bremen, Germany) were used to analyze data and generate ion images.

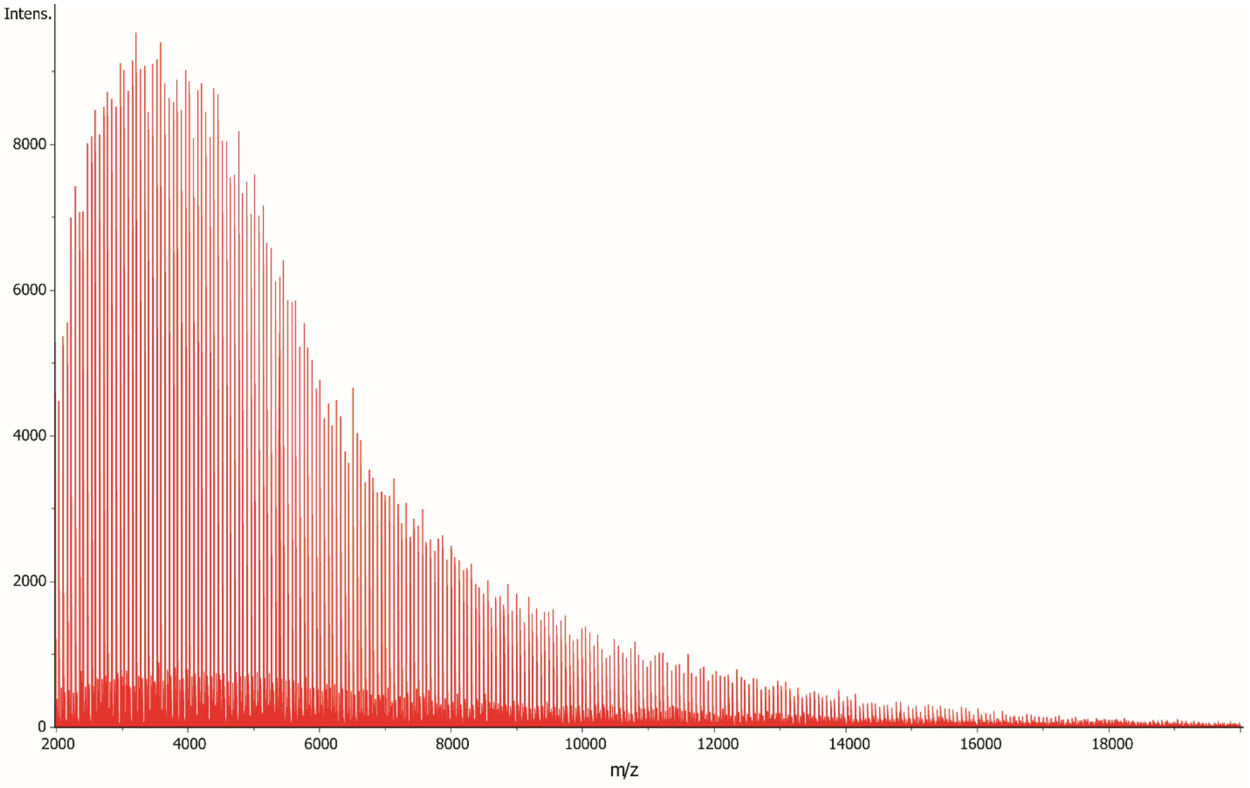

Figure S1. MALDI mass spectrum of red phosphorus.

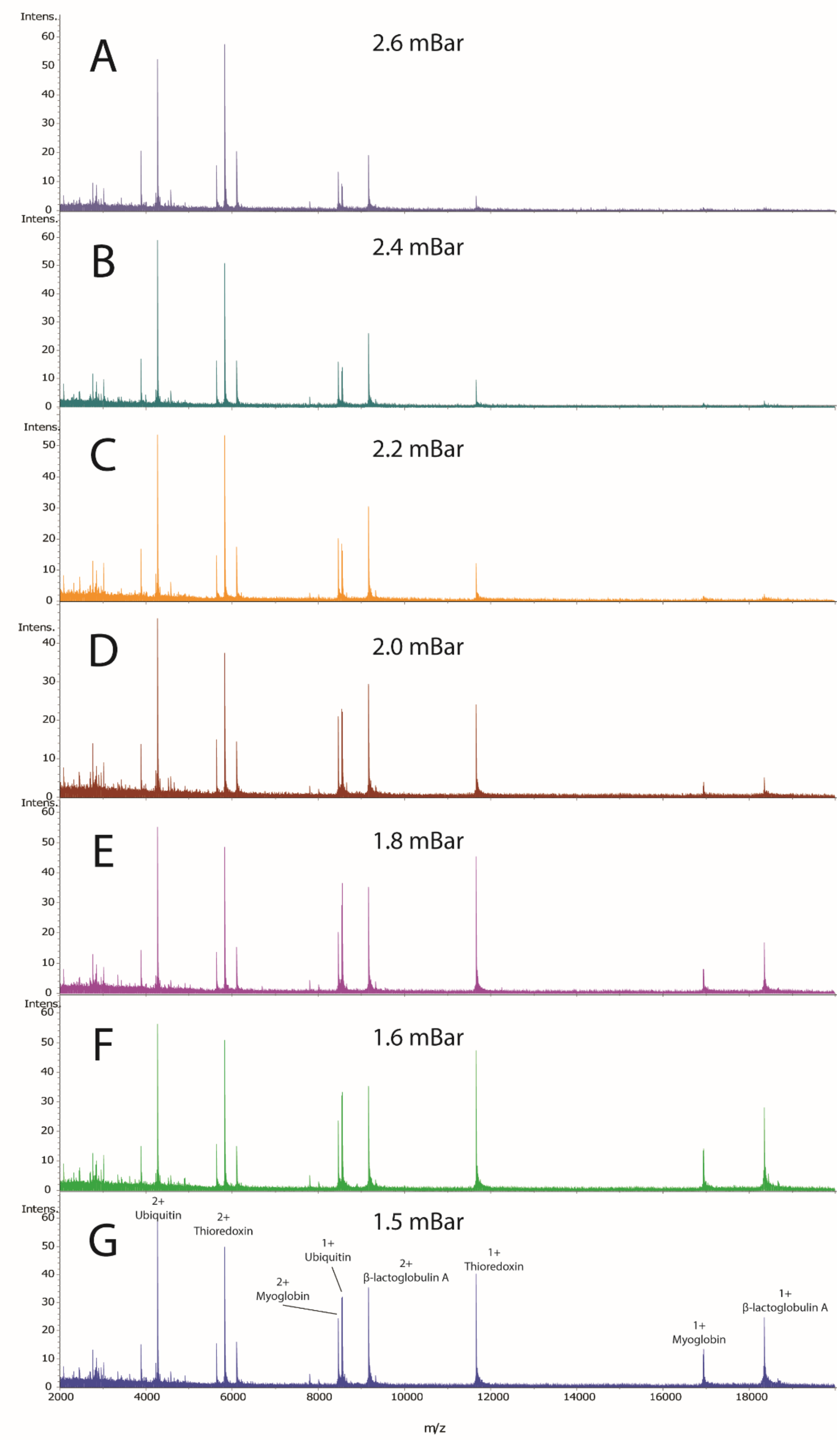

Figure S2. MALDI spectra of the protein standard mixture at a TIMS Tunnel In pressure of 2.6 mBar (A), 2.4 mBar (B), 2.2 mBar (C), 2.0 mBar (D), 1.8 mBar (E), 1.6 mBar (F), and 1.6 mBar (G). Reducing the TIMS Tunnel In pressure increases the intesities of lower charge state ions.

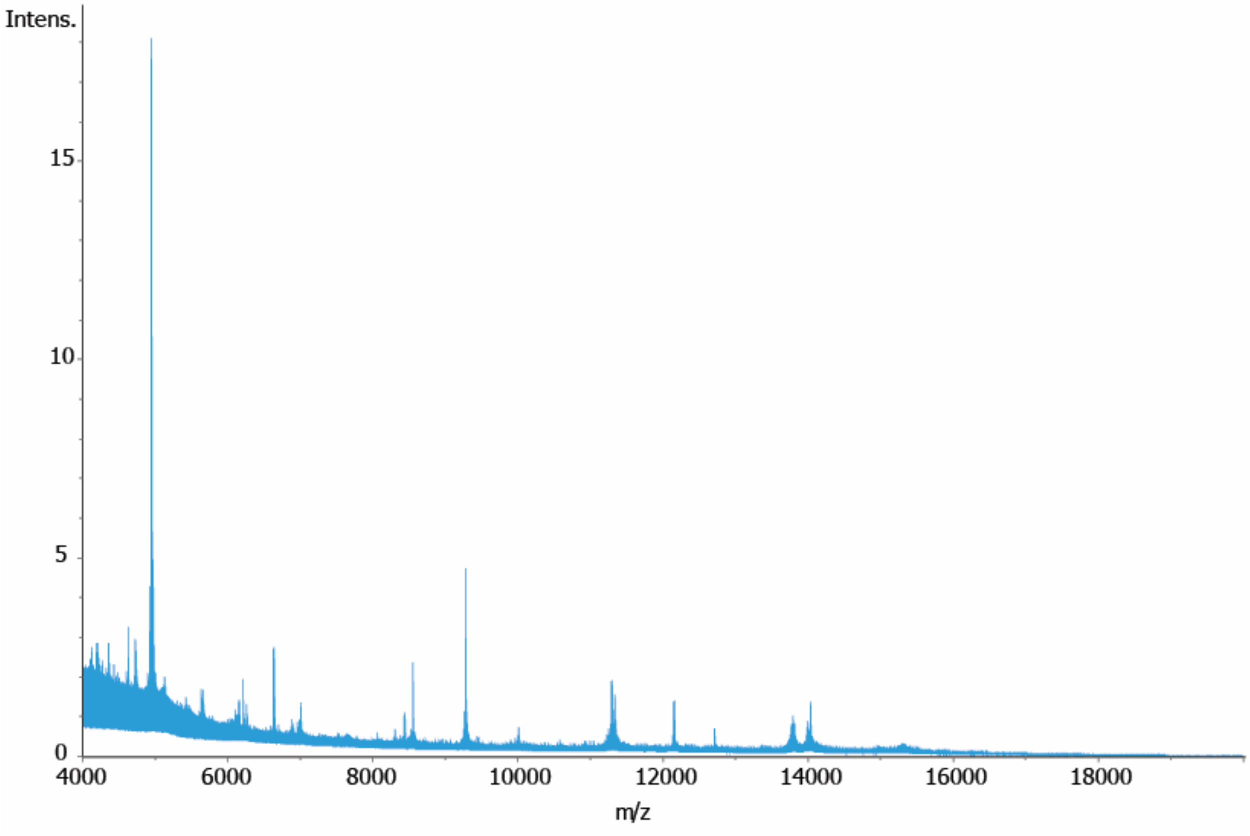

Figure S3. MALDI mass spectrum averaged across the entire mouse pup tissue section.

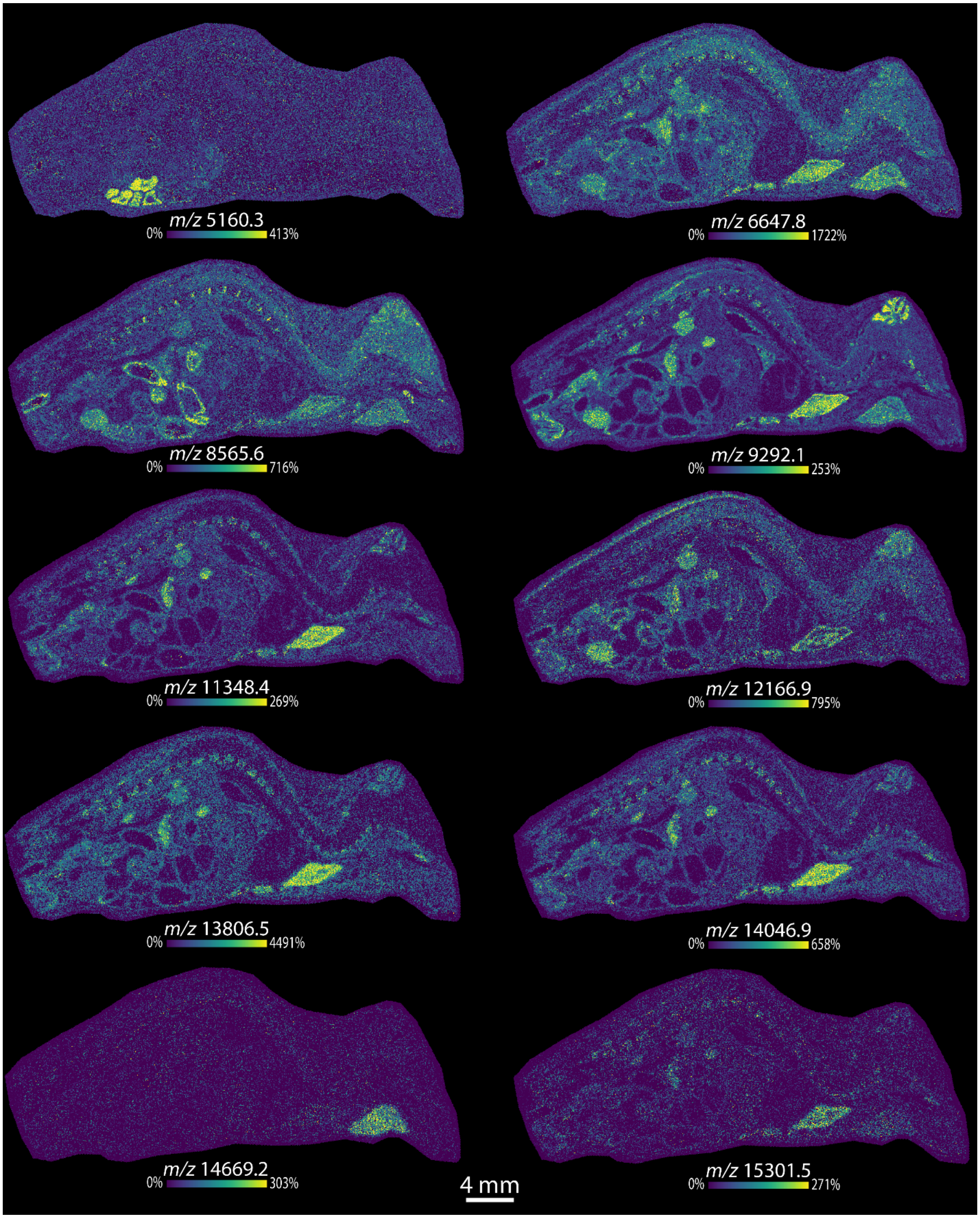

Figure S4. Representative ion images showing localization of protein ions to specific anatomical features.

Table S1. List of red phorphorus-derived *m/z* values used for instrument calibration

#	*m/z*	#	*m/z*
**P5**	154.868	**P59**	1827.451
**P7**	216.816	**P61**	1889.399
**P9**	278.763	**P63**	1951.346
**P11**	340.711	**P65**	2013.294
**P13**	402.658	**P67**	2075.241
**P15**	464.606	**P69**	2137.189
**P17**	526.553	**P71**	2199.137
**P19**	588.501	**P73**	2261.084
**P21**	650.448	**P75**	2323.032
**P23**	712.396	**P77**	2384.979
**P25**	774.343	**P79**	2446.927
**P27**	836.291	**P85**	2632.769
**P29**	898.239	**P95**	2942.507
**P31**	960.186	**P115**	3561.982
**P33**	1022.134	**P135**	4181.457
**P35**	1084.081	**P155**	4800.932
**P37**	1146.029	**P175**	5420.408
**P39**	1207.976	**P195**	6039.883
**P41**	1269.924	**P215**	6659.358
**P43**	1331.871	**P231**	7154.938
**P45**	1393.819	**P251**	7774.414
**P47**	1455.766	**P271**	8393.889
**P49**	1517.714	**P291**	9013.364
**P51**	1579.661	**P311**	9632.839
**P53**	1641.609	**P335**	10376.210
**P55**	1703.556	**P361**	11181.530
**P57**	1765.504	**P403**	12482.430

Table S2. Protein standards

Protein Name	Vendor/ Catalog #	UniProt Accession #	Molecular Formula	Monoisotopic Mass (Da)	Protein Sequence
Ubiquitin	Sigma/U6253	P0CG522	$C_{378}H_{629}N_{105}O_{118}S_1$	8559.62	01 MQIFVKTLTGKTITLEVEPS 20<br>21 DTIENVKAKIQDKEGIPPDQ 40<br>41 QRLIFAGKQLEDGRTLSDYN 60<br>61 IQKESTLHLVLRLRGG
Thioredoxin	Sigma/T0910	P0AA25	$C_{528}H_{836}N_{132}O_{159}S_3$	11,666.06	01 MSDKIIHLTDDSFDTDVLKA 20<br>21 DGAILVDFWAEWCGPCKMIA 40<br>41 PILDEIADEYQGKLTVAKLN 60<br>61 IDQNPGTAPKYGIRGIPTLL 80<br>81 LFKNGEVAATKVGALSKGQL 100<br>101 KEFLDANLA
Apomyoglobin	Sigma/M5696	P68082	$C_{774}H_{1221}N_{211}O_{219}S_3$	16940.96	01 MGLSDGEWQQVLNVWGKVEA 20<br>21 DIAGHGQEVLIRLFTGHPET 40<br>41 LEKFDKFKHLKTEAEMKASE 60<br>61 DLKKHGTVVLTALGGILKKK 80<br>81 GHHEAELKPLAQSHATKHKI 100<br>101 PIKYLEFISDAIIHVLHSKH 120<br>121 PGDFGADAQGAMTKALELFR 140<br>141 NDIAAKYKELGFQG
β-lactoglobulin A	Sigma/L7880	B5B0DA	$C_{821}H_{1322}N_{206}O_{250}S_9$	18,351.42	01 MKCLLLALALTCGAQALIVT 20<br>21 QTMKGLDIQKVAGTWYSLAM 40<br>41 AASDISLLDAQSAPLRVYVE 60<br>61 ELKPTPEGDLEILLQKWEND 80<br>81 ECAQKKIIAEKTKIPAVFKI 100<br>101 DALNENKVLVLDTDYKKYLL 120<br>121 FCMENSAEPEQSLVCQCLVR 140<br>141 TPEVDDEALEKFDKALKALP 160<br>161 MHIRLSFNPTQLEEQCHI

[1]Cysteine residues highlighted in yellow are disulfide bound.
[2]Amino acids highlighted in red are not included in the molecular formulas or the monoisotopic masses and are not part of the detected protein ions.

Equation S1. Equation used to calculate the theoretical acquisition time (T_{aqn}) per spectrum on a FT-ICR mass spectrometer with magnetic field strength (B_0) of 15T at *m/z* 14,000, with a resolution (*m/Δm*) of 40,000.[3]

$$\frac{m}{\Delta m} = \frac{1.274\ x\ 10^7 z\ B_0 T_{aqn}}{m}$$

$$\left(\frac{m}{\Delta m}\right)\frac{m}{1.274\ x\ 10^7 z\ B_0} = T_{aqn}$$

Equation S2. Equation used to calculate the total acquisition time for a MALDI image containing 170,000 pixels, i.e. 170,000 spectra, on a 15T FT-ICR mass spectrometer with a resolving power of 40,000 at *m/z* 14000.

$$T_{aqn}\ \times 170{,}000\ = Total\ aquisition\ time$$

References

(1) Yang, J.; Norris, J. L.; Caprioli, R. Novel Vacuum Stable Ketone-Based Matrices for High Spatial Resolution MALDI Imaging Mass Spectrometry. *Journal of Mass Spectrometry* **2018**, *53* (10), 1005–1012. https://doi.org/10.1002/jms.4277.

(2) Spraggins, J. M.; Djambazova, K. V.; Rivera, E. S.; Migas, L. G.; Neumann, E. K.; Fuetterer, A.; Suetering, J.; Goedecke, N.; Ly, A.; Van De Plas, R.; Caprioli, R. M. High-Performance Molecular Imaging with MALDI Trapped Ion-Mobility Time-of-Flight (TimsTOF) Mass Spectrometry. *Anal Chem* **2019**, *91* (22), 14552–14560. https://doi.org/10.1021/acs.analchem.9b03612.

(3) Marshall, A. G.; Hendrickson, C. L.; Jackson, G. S. Fourier Transform Ion Cyclotron Resonance Mass Spectrometry: A Primer. *Mass Spectrom Rev* **1998**, *17* (1), 1–35. https://doi.org/10.1002/(SICI)1098-2787(1998)17:1<1::AID-MAS1>3.0.CO;2-K.
